# Aromatase Inhibition and Electroconvulsive Seizures in Adolescent Rats: Antidepressant and Long-Term Cognitive Sex Differences

**DOI:** 10.1093/ijnp/pyad047

**Published:** 2023-08-10

**Authors:** Sandra Ledesma-Corvi, M Julia García-Fuster

**Affiliations:** IUNICS, University of the Balearic Islands, Palma, Spain; Health Research Institute of the Balearic Islands (IdISBa), Palma, Spain; IUNICS, University of the Balearic Islands, Palma, Spain; Health Research Institute of the Balearic Islands (IdISBa), Palma, Spain

**Keywords:** ECS, sex-specific differences, estrogens, adolescence, depression

## Abstract

**Background:**

We recently showed sex differences in the antidepressant-like potential of electroconvulsive seizures (ECS) in adolescent rats; whereas it worked for male rats, it was inefficacious in females. Because sex steroids might be important modulators of these sex disparities, we evaluated the role of estrogens in the differential response induced by adolescent ECS. Moreover, given the literature suggesting certain cognitive sequelae from ECS exposure, we aimed at evaluating its long-term safety profile in adulthood.

**Methods:**

Adolescent Sprague-Dawley rats were pretreated with letrozole (1 mg/kg/day) or vehicle (1 mL/kg/day) for 8 days (i.p.) and treated during the last 5 days (3 hours later) with ECS (95 mA, 0.6 s, 100 Hz) or SHAM. Antidepressant-like responses were measured in the forced swim test, and long-term cognitive performance was assessed in the Barnes maze.

**Results:**

During adolescence, whereas ECS alone exerted an antidepressant-like response in male rats, its combination with letrozole permitted ECS to also induce efficacy in females. Moreover, adolescent ECS treatment improved cognitive performance in adulthood although exclusively in male rats.

**Conclusions:**

Adolescent ECS demonstrated an antidepressant-like potential together with certain long-term beneficial cognitive effects but exclusively in male rats. For females, efficacy was restricted to a situation in which the biosynthesis of estrogens was reduced. Therefore, estrogens and/or testosterone levels play a crucial role in the sex disparities induced by ECS in Sprague-Dawley rats. Based on this study and on the literature supporting its safety, ECS should be encouraged for use in cases of treatment-resistant depression during adolescence, while adhering to sex-specific considerations.

**Significance Statement**

The induction of electroconvulsive seizures (ECS) is a safe therapeutic option for adolescents with treatment-resistant depression. However, given the differences in efficacy induced by sex and age, there is an urgent need to further characterize the effects of ECS in adolescence. This study proves clear sex differences in the antidepressant-like response induced by ECS in adolescent Sprague-Dawley rats, with male rats benefiting more from the treatment, through an antidepressant-like response in adolescence and certain long-term improvements in cognitive performance in adulthood. Moreover, although ECS did not show an antidepressant-like response in female adolescent rats, its combination with letrozole potentiated an antidepressant-like response, suggesting a role for the biosynthesis of estrogens and/or for the accumulation of testosterone in the therapeutic response that deserves future studies. Based on this study and on the literature supporting its safety, ECS should be encouraged to use in cases of treatment-resistant depression during adolescence while adhering to sex-specific considerations.

## INTRODUCTION

There is an urgent need to provide novel treatment strategies for adolescents with treatment-resistant depression because therapeutical options for these young patients are lacking and the pharmacological alternatives either fail to respond (e.g., Zhou et al., 2014), display lower efficacy (Bylund and Reed, 2007; Bis-Humbert et al., 2020, 2021; García-Cabrerizo et al., 2020), and/or could even generate a harmful response (i.e., increased suicide ideation; Cipriani et al., 2016). In this scenario, electroconvulsive therapy (ECT) offers a safe and effective treatment for mood disorders in adolescents (Ghaziuddin and Walter, 2014; Weiner and Reti, 2017; Karayağmurlu et al., 2020; Døssing and Pagsberg, 2021; Castaneda-Ramirez et al., 2022; Chen et al., 2022; Yu et al., 2023). Although it is less commonly applied, in the recent years there has been a general recommendation among clinicians for ECT to be considered and broadly used, especially in severe and treatment-refractory cases for adolescence. In any case, ECT parameters must be adjusted for age and sex (e.g., Rasimas et al., 2007; Peterchev et al., 2010; Güney et al, 2020; Blanken et al., 2023) because the electrical charge needed to induce an effective convulsion is lower for females (as opposed to males) and younger age (e.g., Salvador Sánchez et al., 2017).

In this context, our research group has been using experimental rodent models to characterize the potential sex- and age-specific differences in the antidepressant-like response induced by electroconvulsive seizures (ECS) (e.g., García-Fuster and García-Sevilla, 2016; García-Cabrerizo et al., 2020; Ledesma-Corvi and García-Fuster, 2023). In particular, repeated ECS (95 mA for 0.6 seconds at a frequency of 100-Hz square wave pulses, pulse width 0.6 milliseconds, 5 days, 1 shock per day) induced an antidepressant-like response in Sprague-Dawley male rats in both adolescence (García-Cabrerizo et al., 2020) and adulthood (García-Fuster and García-Sevilla, 2016; García-Cabrerizo et al., 2020). However, in female adolescent rats, ECS was ineffective and/or deleterious with the same dose parameters as used in male rats (García-Cabrerizo et al., 2020) but also when lowering the intensity of the pulse applied during ECS from 95 mA to 75 or 55 mA (Ledesma-Corvi and García-Fuster, 2023). Interestingly, 55 and 75 mA of current applied during ECS induced an antidepressant-like effect in adult female rats (Ledesma-Corvi and García-Fuster, 2023), therefore suggesting a decreased sensitivity to ECS during adolescence for this particular sex. The disparities in efficacy observed between sexes might be influenced by a variety of factors, including the level of sex steroids, such as estrogens and testosterone, both in the periphery and locally in the brain (e.g., Ponton et al., 2022) and reinforce the need to include both sexes in all preclinical studies (Miller et al., 2017; Beltz et al., 2019; Docherty et al., 2019).

In this context, prior studies have reported the possible role of estrogens in the sex-specific disparities in depression (e.g., Hwang et al., 2020; Pavlidi et al., 2021) and the efficacy of certain antidepressants (e.g., Berlanga and Flores-Ramos, 2006; Martínez-Mota et al., 2008; Damoiseaux et al., 2014; Pavlidi et al., 2021). However, to the best of our knowledge, there is no prior literature on how the levels of estrogens might affect the antidepressant-like response exerted by ECS. In this context, the use of letrozole, a pharmacological option that inhibits the aromatase involved in the biosynthesis of estrogens from androgens (e.g., Simpson et al., 2002), was previously used in the context of characterizing sex-related antidepressant-like responses induced by different drugs in rats (e.g., see Kokras et al., 2014 for fluoxetine; see Ledesma-Corvi et al., 2023 for our own studies with subanesthetic doses of ketamine). Therefore, in the present study, we used male and female adolescent Sprague-Dawley rats to describe the antidepressant-like response of ECS while inhibiting the biosynthesis of estrogens with letrozole.

Moreover, ECS is usually associated with the development of certain cognitive side effects (i.e., anterograde amnesia; see revisions throughout the years by Devanand et al., 1994; Reid and Stewart, 1997; Andrade et al., 2008), although there is an open debate about whether these cognitive effects might be linked to the anesthetic used to apply the treatment because no consensus exists regarding the optimal anesthetic drug to use (e.g., Soehle and Bochem, 2018). In fact, there are many studies associating the therapeutic outcome in patients undergoing this procedure with the use of different anesthetics. For example, when comparing ketamine with thiopental, it was suggested that ketamine administration during treatment was well tolerated and patients experienced earlier improvements in depressive symptoms, longer seizure duration, and better cognitive performance compared with thiopental (Yoosefi et al., 2014). On a different note, thiopental led to better seizure duration and quality but showed a greater risk of cognitive adverse effects compared with propofol (Kavakbasi et al., 2022). Thus, in this study, in an attempt to characterize the long-term safety profile of adolescent ECS and because there was no anesthetic present, we also aimed to evaluate cognitive performance and affective-like response later on in adulthood.

## MATERIALS AND METHODS

### Animals

A total of 106 adolescent Sprague-Dawley rats (59 male and 47 female), bred in the animal facility at the University of the Balearic Islands, were used in this study. Rats were housed in a controlled environment (22°C, 70% humidity, 12-hour-light/-dark cycle) in regular cages (2–4 rats per cage) with unlimited access to a standard diet and water. Before any procedures, rats were handled by the experimenter to reduce future stress and/or suffering. All procedures were approved by the local bioethical committee (University of the Balearic Islands) and the regional government (Conselleria Medi Ambient, Agricultura i Pesca, Direcció General Agricultura i Ramaderia, Govern de les Illes Balears) following ARRIVE guidelines (McGrath and Lilley, 2015) and EU Directive 2010/63/EU of the European Parliament and of the Council. Although body weight was monitored across time, we did not check the specific stages of the estrous cycle. This was justified because female rats do not seem to be more variable than male rats in neuroscience research due to hormonal periodicity (e.g., Becker et al., 2016; Kaluve et al., 2022) but mainly because the cyclicity of females was not part of our research question (see Beltz et al., 2019).

### Adolescent Treatments

**Figure 1.**
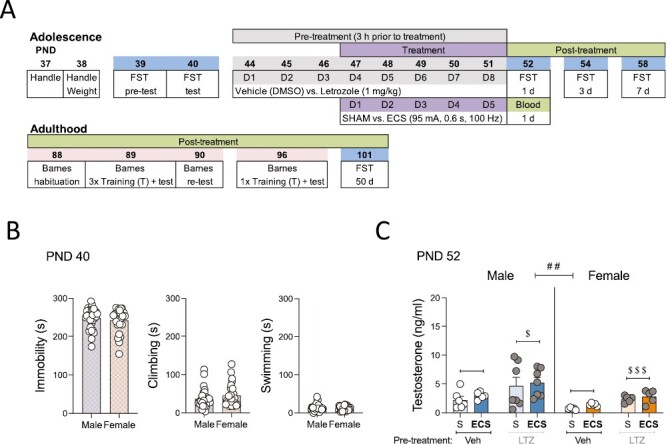
(A) Experimental timeline. Aromatase inhibition and electroconvulsive seizures in adolescent rats: antidepressant and long-term cognitive sex differences. D, day of treatment; d, day post treatment; ECS, electroconvulsive seizures; FST, forced swim test; PND, postnatal day. (B) Baseline behavioral responses in the forced swim test on PND 40. Time (seconds) spent immobile, climbing or swimming before adolescent treatment in the forced swim test. Data represent mean ± SEM of the time (seconds) spent in each behavior. Individual values are shown for each rat (symbols). No changes were observed between sexes (Student *t* test). (C) Testosterone levels in plasma following ECS and/or letrozole administration on PND 52. Testosterone levels (ng/mL) as measured 1 day post-ECS treatment in adolescent male and female rats. Data represent mean ± SEM of testosterone levels (ng/mL). Individual values are shown for each rat (symbols). A 3-way ANOVA (independent variables: sex, pretreatment. and treatment) or 2-way ANOVAs for each sex separately (independent variables: Pretreatment and treatment) were performed and statistical results are shown in supplementary Table 1. ^##^*P < *.01 comparing female vs male rats (effect of Sex). ^$$$^*P* < .001 and ^$^*P < *.05 comparing letrozole vs vehicle pretreated rats (effect of pretreatment). LTZ, letrozole; S, SHAM; Veh, vehicle.

All male and female adolescent rats were pretreated (i.p.) with letrozole (1 mg/kg/day) or vehicle (1 mL/kg/day dimethyl sulfoxide) for 8 consecutive days, followed 3 hours later by a daily session of ECS (95 mA for 0.6 seconds at a frequency of 100-Hz square wave pulses, pulse width 0.6 milliseconds) via earclip electrodes and using a pulse generator (ECT Unit 7801, Ugo Basile, Italy), or SHAM control (without electrical current), for 5 consecutive days (days 4–8 of letrozole treatment; see Figure 1). The parameters of ECS were based on our prior studies that reliably reproduce the characteristic tonic and clonic convulsions (García-Fuster and García-Sevilla, 2016; García-Cabrerizo et al., 2020). Some of these rats were used to quantify testosterone levels in plasma (26 male and 20 female), whereas the rest were exposed to behavioral phenotyping in adolescence and later on in adulthood through the forced swim test and Barnes maze test (33 males and 27 females).

### Hormonal Assay

For ascertaining the effects of letrozole on testosterone levels in plasma, the experimental groups were as followed: vehicle-SHAM (male: n = 6; female: n = 5), vehicle-ECS (male: n = 6; female: n = 5), letrozole-SHAM (male: n = 7; female: n = 5), letrozole-ECS (male: n = 7; female: n = 5). Following treatment, trunk blood samples were collected 1 day post treatment (Figure 1). Plasma was recovered by centrifuging blood samples for 15 minutes (1500 × g at 4°C) and was then stored at −80°C until the levels of accumulated testosterone were quantified by a standard ELISA assay (LDN, AR E-8000R, Nordhorn, Germany), whose sensitivity was 0.066 ng/mL, and as previously characterized by our group (Ledesma-Corvi et al., 2023).

### Behavioral Phenotyping: Forced Swim Test and Barnes Maze Test

For behavioral phenotyping, the experimental groups per sex were as follows: vehicle-SHAM (male: n = 8; female: n = 7), vehicle-ECS (male: n = 9; female: n = 7), letrozole-SHAM (male: n = 8; female: n = 7), and letrozole-ECS (male: n = 8; female: n = 6). The potential effects of ECS, letrozole, and/or their combination during adolescence and later on in adulthood were screened under the stress of the forced swim test (Slattery and Cryan, 2012) following a standardized protocol in our group for characterizing antidepressant-like responses (e.g., García-Fuster and García-Sevilla, 2016; Bis-Humbert et al., 2020; García-Cabrerizo et al., 2020; Ledesma-Corvi and García-Fuster, 2022; Ledesma-Corvi et al., 2022). Rats were individually placed in water (25ºC ± 1ºC) tanks (41 cm high × 32 cm diameter, 25 cm depth) for 15 minutes (pretest session, postnatal day [PND] 39) followed by 5-minute test sessions that were videotaped (see Figure 1). Test sessions, which were recorded, were repeated across time to evaluate the progression of the response: baseline (PND 40; see Figure 1B and supplementary Table 1 for immobility, climbing, and swimming rates for male and female rats before any treatment), repeated treatment effects in adolescence (1, 3, and 7 days post treatment; PND 52, 54, and 58, respectively), and potential long-term effects in adulthood (50 days post treatment; PND 101). Videos were later analyzed by an experimenter blinded to the treatment groups to determine individual immobility vs active behaviors (climbing or swimming) for each rat (Behavior Tracker, CA, USA).

To evaluate the possible long-term effect of the adolescent treatment (ECS, letrozole, or their combination) on cognition, we relied on the Barnes maze test, which was originally developed to test hippocampal-dependent spatial learning and memory (Rosenzweig et al., 2003). The Barnes maze used in this experiment consisted of a circular platform with 18 holes equidistantly located around the perimeter and with a black escape box or target below one of them. Moreover, extra-maze cues were all around the room as a reference to learn the position of the target box, and a bright light was used as an aversive stimulus to find the target because it accentuated the natural agoraphobia of rats. The first day (PND 88, 37 days post treatment), rats were habituated to the maze by placing them in a black start chamber located in the middle of the maze under a bright light (500 W). After 10 seconds, the chamber was lifted and rats were allowed to find and enter the black escape box for 3 minutes. On test day (PND 89, 38 days post treatment), each rat first performed 3 training trials (separated 10 minutes) that finished either when the animal entered the target or after 3 minutes, when it was manually placed into the target box and was left there for 1 minute to habituate. Then, 10 minutes later, the actual test occurred by allowing rats to freely explore the maze for 90 seconds to find the target box. This test was repeated 24 hours later on PND 90 (Figure 1). Finally, a week later (PND 96, 45 days post treatment), the target box was changed and rats were exposed to a new test session 10 minutes after a single trial (3 minutes of exploration to find the target box), in which animals were allowed to freely explore the maze for 90 seconds to find the new target box. The amount of time spent (s) to resolve the maze, as well as the number of errors committed and the strategy used (e.g., direct, serial, mixed), were used as a measure of spatial working memory performance (e.g., Hernández-Hernández et al., 2018).

### Data Analysis and Statistics

Data were analyzed with GraphPad Prism, Version 10 (Beta) (GraphPad Software, San Diego, CA, USA), and results are presented as mean values ± SEM while including individual symbols for each rat, as suggested by the guidelines for displaying data and statistical methods in experimental pharmacology (e.g., Curtis et al., 2018; Michel et al., 2020). Basal behavioral responses in the forced swim test (i.e., immobility, climbing, swimming) were compared between sexes with a Student’s *t* test. Each set of data was evaluated with 3-way ANOVAs (independent variables: sex, pretreatment, and treatment). When sex differences emerged, data were also analyzed for each sex separately by 2-way ANOVAs (independent variables: pretreatment and treatment). Multiple comparisons were performed with either Sidak’s or Tukey’s test when appropriate. The level of significance was fixed at *P* ≤ .05.

## RESULTS

### Testosterone Levels in Plasma Following Letrozole Administration in Adolescence

When analyzing the effects of inhibiting the biosynthesis of estrogens through the pharmacological administration of letrozole (an aromatase inhibitor), the results showed, as expected, a significant overall sex difference in testosterone levels (ng/ml) (Figure 1C; supplemental Table 1), with female rats displaying lower levels (−1.93 ng/mL of testosterone, ^##^*P* = .003) than males. Therefore, the effects of letrozole pretreatment were evaluated for each sex separately, rendering significant effects for both male (+2.33 ±  1.03 ng/mL; ^*$*^*P = *.035 vs vehicle) and female (+1.52 ±  0.30 ng/mL; ^*$$$*^*P < *.001 vs vehicle) rats (Figure 1C). No changes in testosterone levels were produced by adolescent ECS treatment (see supplementary Table 1).

### Antidepressant-Like Effects of Adolescent ECS in Letrozole Pretreated Rats

**Figure 2.**
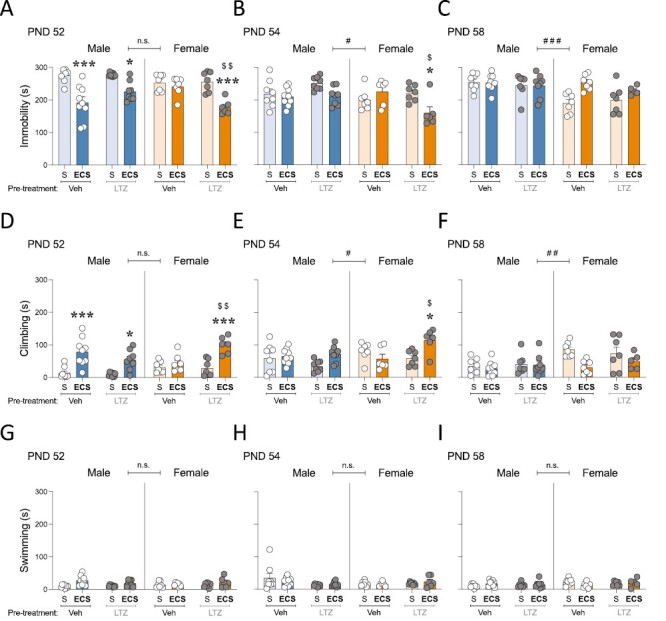
Evaluating the antidepressant-like effects of electroconvulsive seizures (ECS) and/or letrozole (LTZ) in adolescent male and female rats. (A–C) Time spent (seconds) immobile, (D–F) climbing, or (G–I) swimming in the forced swim test as measured 1, 3, and 7 days post-ECS treatment (postnatal day [PND] 52, 54, and 58 respectively) in adolescent male and female rats pre-treated with vehicle or letrozole. Data represents mean ± SEM of the time (s) spent in each behavior. Individual values are shown for each rat (symbols). Three-way ANOVAs (independent variables: sex, pretreatment, and treatment) or 2-way ANOVAs (independent variables: pretreatment and treatment) were performed and results are shown in supplementary Table 1. ^###^*P < *.001, ^##^*P < *.01, and ^#^*P < *.05 comparing female vs male rats (effect of sex). ***P < .001 and *P < .05 comparing ECS vs SHAM groups. ^$$^*P < *.01 and ^$^*P < *.05 comparing LTZ-ECS vs Veh-ECS groups. S, SHAM; Veh, vehicle.

Changes in immobility were evaluated during adolescence at 1, 3, and 7 days post-ECS treatment in the forced swim test as a measure of an antidepressant-like response. On PND 52 (1 day post treatment), a 3-way ANOVA detected a significant sex × pretreatment × treatment interaction (see supplementary Table 1). Sidak’s multiple comparisons detected several significant changes. In particular, in male rats, ECS induced an antidepressant-like effect during adolescence independently of pretreatment (−84 ± 15 seconds, ***P < .001 and −52 ± 15 seconds, *P = .018 vs SHAM pretreatment group; Figure 2A). However, although in female rats ECS alone did not induce an antidepressant-like effect (−11 ± 16 seconds, *P* = .999 when comparing vehicle-ECS vs vehicle-SHAM), it did when rats were pretreated with letrozole (−79 ± 17 seconds, ***P < .001 vs letrozole-SHAM; −64 ± 17 seconds, ^$$^*P = *.005 vs vehicle-ECS; Figure 2A). On PND 54 and PND 58 (3 and 7 days post treatment, respectively), no sex × pretreatment × treatment interactions were observed (see supplementary Table 1). However, because noticeable sex differences emerged at these time points of analysis (PND 54: ^#^*P* = .018 and PND 58: ^###^*P* < .001; see supplementary Table 1), the combined effects of letrozole pretreatment and ECS treatment were analyzed for each sex separately through 2-ways ANOVAs (supplemental Table 1; Figure 2B and C). For male rats, there was an overall effect of ECS treatment on PND 54 (−24 ± 11 seconds, *P = *.030 vs SHAM-treated rats; Figure 2B) that dissipated on PND 58 (Figure 2C). Interestingly, for female rats, there was a pretreatment × treatment interaction on PND 54 (supplementary Table 1), and Tukey’s multiple comparisons test revealed that the antidepressant-like effects observed in letrozole-ECS rats were still present 3 days post treatment (−61 ± 19 seconds, *P = .024 vs letrozole-SHAM; −65 ± 20 seconds, $*P = *.019 vs vehicle-ECS; Figure 2B). On PND 58 (7 days post treatment), the beneficial effects of ECS dissipated, because the significant effect of treatment observed (supplementary Table 1) was driven by an overall increase in immobility in ECS-treated groups (+46 ± 12 seconds, *P = *.001 vs SHAM-treated rats; Figure 2C).

Similar results, although in the opposite direction, were obtained when analyzing climbing behavior because the beneficial effects induced by ECS were mediated by increases in climbing (see Figure 2D and E; supplementary Table 1). Therefore, ECS increased climbing behavior in adolescent male rats at 1 day (+65 ± 13 seconds, ***P < .001 and +44 ± 13 seconds, *P = .020 vs SHAM pretreatment group; Figure 2D) and up to 3 days post treatment (+22 ± 10 seconds, *P = *.030 vs SHAM-treated rats and independently of pretreatment; Figure 2E). However, in female rats, ECS increased climbing behavior, but only when rats were pretreated with letrozole, which was observed at 1 day (+71 ± 15 seconds, ***P < .001 vs letrozole-SHAM; +57 ± 15 s, $$*P = *.004 vs vehicle-ECS; Figure 2D) and up to 3 days post treatment (+56 ± 17 seconds, *P = .015 vs letrozole-SHAM; +58 ± 17 seconds, $*P = *.015 vs vehicle-ECS; Figure 2E). Moreover, at 7 days post treatment, ECS decreased climbing behavior in female rats independently of pretreatment (−39 ± 13 seconds, *P = *.007 vs SHAM-treated rats; Figure 2F). Finally, no changes were observed in swimming behavior (see Figure 2G–I; supplementary Table 1).

### Long-Term Effects of Adolescent ECS Treatment in Adult Rats

**Figure 3.**
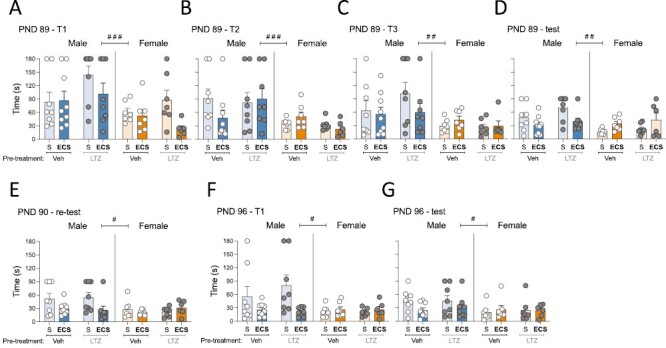
Evaluating the long-term effects on cognitive performance in adulthood following adolescent electroconvulsive seizures (ECS) and/or letrozole (LTZ) treatments in male and female rats. Time spent (seconds) in (A–C) 3 consecutive trials (T1, T2, T3) and (D–E) 2 test sessions (spaced 24 hours, test on postnatal day [PND] 89 and retest on PND 90) to complete the Barnes maze. Time spent (seconds) in (F) a new trial (changing the location of the target box on PND 96-T1) and (G) test session (PND 96) to complete the Barnes maze 1 week after. Data represent mean ± SEM of the time (seconds) spent to complete the Barnes maze. Individual values are shown for each rat (symbols). Three-way ANOVAs (independent variables: sex, pretreatment, and treatment) or 2-way ANOVAs (independent variables: pretreatment and treatment) were performed and results are shown in supplementary Table 2. ^###^*P < *.001, ^##^*P < *.01, and ^#^*P < *.05 comparing female vs male rats (effect of Sex). S, SHAM; Veh, vehicle.

The potential long-term effects on cognition and affect induced by adolescent ECS and/or letrozole were evaluated in adulthood in the Barnes maze test (38–45 days post treatment; PND 89–96) and the forced-swim test respectively (50 days post treatment; PND 101; Figure 1A). In particular, clear sex differences were observed when measuring the time rats needed to resolve the Barnes maze during both training and/or test sessions (see supplementary Table 2), with female rats needing significantly less time to resolve the Barnes maze than males (Figure 3). Therefore, results were analyzed through 2-way ANOVAs for each sex separately and including pretreatment and treatment as independent variables. From this later analysis, the main results reported no long-term effects driven by the adolescent pretreatment (vehicle vs letrozole) except for female rats on training session 2 on PND 89 (supplementary Table 2), which showed faster overall times to resolve the maze for letrozole pretreated rats (−15 ± 7 seconds, *P = *.039 vs vehicle pretreated female rats; Figure 3B). Moreover, some significant effects of treatment were observed during test days (see supplementary Table 2), particularly on PND 89; whereas adolescent ECS improved the performance in the Barnes maze for male rats (−24 ± 9 seconds, *P = *.011 vs SHAM-treated male rats), it worsened for female rats (+19 ± 8 seconds, *P = *.020 vs SHAM-treated female rats; Figure 3D). The beneficial long-term effects of adolescent ECS in male rats were still observed on PND 90 (retest: −25 ± 9 seconds, *P = *.012 vs SHAM-treated male rats; Figure 3E) and on PND 96 (new training session with changed settings: −45 ± 16 seconds, *P = *.009; Figure 3F; test session: −17 ± 9 seconds, *P = *.067; Figure 3G). No changes were observed at those time points for female rats (Figure 3E–G). Besides the time used to resolve the Barnes maze, we also measured the number of errors made while trying to resolve it. The results demonstrated no major significant changes by sex, pretreatment, or treatment (supplementary Figure 2 and Table 2). Moreover, supplementary Figure S3 shows a qualitative approximation (not quantified due to small sample size for this type of analysis) representing the percent strategy (direct, serial, mixed, not completed) used to resolve the maze for each treatment group and training or test session.

**Figure 4.**
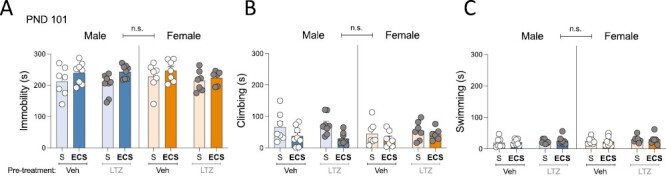
Evaluating the long-term effects in the forced swim test in adulthood following adolescent electroconvulsive seizures (ECS) and/or letrozole treatments in male and female rats. (A) Time spent (seconds) immobile, (B) climbing, or (C) swimming in the forced swim test as measured 50 days post treatment (postnatal day [PND] 101). Data represent mean ± SEM of the time (seconds) spent in each behavior. Individual values are shown for each rat (symbols). Three-way ANOVAs (independent variables: sex, pretreatment, and treatment) or 2-way ANOVAs (independent variables: pretreatment and treatment) were performed and results are shown in supplementary Table 2. LTZ, letrozole; S, SHAM; Veh, vehicle.

Finally, adolescent ECS, compared with SHAM-treated rats, induced long-term changes in the response measured in the forced swim test (Figure 4; supplementary Table 2) because it increased immobility (+26 ± 9 seconds; *P* = .009; Figure 4A) and decreased climbing (−26 ± 8 seconds; *P* = .003; Figure 4B) independently of sex and/or pretreatment, whereas it did not affect swimming behavior (Figure 4C). These changes seemed to be driven by a decrease in immobility in SHAM-treated rats as opposed to a worse performance in the test caused by adolescent ECS treatment (see supplementary Figure 3).

## DISCUSSION

The present study demonstrated that the production of estrogens has a crucial role in the sex disparities induced by ECS in terms of an antidepressant-like response in Sprague-Dawley rats. In particular, although ECS (at the parameters tested) was efficacious after a repeated paradigm exclusively in male adolescent rats, its combination with letrozole, an aromatase inhibitor, permitted ECS to generate an antidepressant-like response in female adolescent rats. Moreover, although decreasing the biosynthesis of estrogens with letrozole did not induce by itself any major behavioral responses in adolescent and/or adult rats, it produced the expected significant increases in testosterone levels in adolescence. Finally, some long-term beneficial effects of adolescent ECS treatment were present in adulthood at the level of cognitive performance, showing improved responses in the Barnes maze over time, although only in male rats. In conjunction, the present results demonstrated sex disparities in the effects induced by ECS, with an antidepressant-like potential during adolescence together with certain long-term beneficial effects in adulthood for male rats and with certain antidepressant-like efficacy for female rats, although restricted to a situation in which the biosynthesis of estrogen was reduced.

The present results showed clear and interesting sex differences in the behavioral actions exerted by repeated ECS in adolescence. Particularly, whereas adolescent ECS induced an antidepressant-like response in male rats (i.e., decreased immobility and increased climbing behavior), it was inefficacious in female rats. These observations replicated our prior findings in which the same paradigm of ECS induced a similar course of effects in adolescence (see García-Cabrerizo et al., 2020 and references within). The lack of efficacy in females could be caused, as suggested in the prior literature, by the fact that females might require a lower electrical charge for ECS to induce an effective convulsion (e.g., Salvador Sánchez et al., 2017). In this context, in a separate study we recently demonstrated that lowering the intensity of the daily pulse applied during ECS (from 95 mA to 55 or 75 mA) was capable of inducing an antidepressant-like effect in female adult rats (Ledesma-Corvi and García-Fuster, 2023), which otherwise was lacking (ineffective dose of 95 mA in females, but effective in males; see García-Cabrerizo et al., 2020). However, still no beneficial response was observed at any of the intensity doses tested in adolescent female rats, which might be explained by age differences in the characteristics of the seizures induced by ECS in adolescence compared with adulthood (see Ledesma-Corvi and García-Fuster, 2023).

In searching for mechanisms behind these sex-related disparities, the level of estrogens appeared to be critical mediators in the differential efficacy of certain antidepressants (e.g., Berlanga and Flores-Ramos, 2006; Pavlidi et al., 2021), although to the best of our knowledge no prior studies evaluated the role of estrogens in the antidepressant-like response of ECS. In this context, we relied on letrozole, an aromatase inhibitor used in the context of other antidepressants in adult rats (e.g., see Kokras et al., 2014; see Ledesma-Corvi et al., 2023), to test how it affected the behavioral response of ECS in male and female adolescent rats. In fact, and in line with prior results (Kokras et al., 2014, 2018), adult letrozole administration induced an antidepressant-like effect that parallel an increased in testosterone levels 1 hour post treatment but exclusively in female rats (Ledesma-Corvi et al., 2023), similarly to the effects induced by the administration of repeated testosterone (Frye and Walf, 2009). However, the effects of the same paradigm of letrozole administration during adolescence, as reported in this study, were a bit different because letrozole by itself did not induce an antidepressant-like response but, as expected, significantly increased the levels of plasma testosterone for both sexes.

In regard to the antidepressant-like effects of ECS in letrozole pretreated male and female adolescent rats, the results showed that although letrozole did not further improve the response of ECS in male rats, suggesting probably a maximum effect in the assay evaluated, it permitted ECS to induce an antidepressant-like effect in female rats, which was observed up to 3 days post treatment in the forced swim test. These behavioral effects dissipated over time because no interaction was observed in the forced swim test in adulthood 50 days post treatment. In any case, the adolescent results are really remarkable in the sense that a pharmacological modulation was capable of improving the outcome of ECS for female adolescent rats, which were unresponsive even at lower dose intensities, at which adult females responded (Ledesma-Corvi and García-Fuster, 2023). Moreover, although the beneficial effects could not be exclusively ascertained to increased testosterone levels, because letrozole-SHAM female rats also showed increased levels but lacked an antidepressant-like response, the data provided a mode to improve the therapeutical outcome of ECS in a generally unresponsive sex at a sensitive age period. Future studies will center on evaluating the role of other hormones and/or the potential mechanism by which letrozole interacts with ECS to potentiate its antidepressant-like response in adolescent female rats. For example, and as discussed in the context of other antidepressants effects (e.g., Ledesma-Corvi et al., 2023), letrozole and ECS could modulate common molecular pathways in a synergic way and/or affecting the pharmacodynamic profiles of ECS, producing the enhanced behavioral response in females during adolescence.

As for the long-term impact on cognition caused by adolescent ECS, the present data provided an indication of certain benefits for male rats, because shorter times were observed when resolving the Barnes maze; this is a sign of better spatial learning and memory performance. However, no benefits were observed for female rats, which might be related to the lack of effects induced by ECS in adolescence and because letrozole by itself did not induce any long-term effects. These results provided novel data on the potential long-terms benefits of initiating ECS treatment early during adolescence. Most of the prior published studies that evaluated the negative impact of ECS on cognition were centered on the short-term effects emerging right after adult treatment (e.g., Devanand et al., 1994; Reid and Stewart, 1997; Andrade et al., 2008). Therefore, the present study provided relevant knowledge, which together with reports that concluded that this therapeutical approach was safe and effective for the treatment of mood disorders in the adolescent population (e.g., Karayağmurlu et al., 2020; Døssing and Pagsberg, 2021; Castaneda-Ramirez et al., 2022; Yu et al., 2023), strengthened the general recommendation of considering this treatment approach for a more general use in severe and treatment-refractory cases for adolescence.

Overall, these findings have proven clear sex differences in the antidepressant-like response induced by ECS in adolescent rats, with male rats benefiting more from the treatment both through an antidepressant-like response in adolescence and through certain long-term improvements in cognitive performance in adulthood. Moreover, although ECS did not show an antidepressant-like response in female adolescent rats (at least with the dose parameters tested), its combination with letrozole potentiated an antidepressant-like response, suggesting a role for the biosynthesis of estrogens and/or for the accumulation of testosterone in the therapeutic response that deserves future studies. Based on this study and on the literature supporting its safety, ECS should be encouraged to use in cases of treatment-resistant depression during adolescence while adhering to sex-specific considerations.

## Supplementary Material

pyad047_suppl_Supplementary_Materials
