# Supplementary material for: Aromatase Inhibition and Electroconvulsive Seizures in Adolescent Rats: Antidepressant and Long-Term Cognitive Sex Differences
Source: Int J Neuropsychopharmacol. 2023 Aug 10;26(9):607–15. doi: 10.1093/ijnp/pyad047 (PMC10519810; doi:10.1093/ijnp/pyad047)

Supplementary Materials

Supplementary Table S1. Statistical analysis performed during adolescence. This table represents the statistical analysis performed for each one of the figures shown in the manuscript. Green-shadow boxes represent statistically significant effects.

Adolescence

Figure 1B. Basal FST - PND 40

Unpaired *t* test	Sex
Immobility (s)	*t* = 0.88, *df* = 58, *p* = 0.381
Climbing (s)	*t* = 1.26, *df* = 58, *p* = 0.212
Swimming (s)	*t* = 1.08, *df* = 58, *p* = 0.285

Figure 1C. Testosterone (ng/ml)

Three-way ANOVA: F (DFn, DFd), *p* value	Sex	Pre-treatment (Veh vs. LTZ)	Treatment (SHAM vs. ECS)	Sex x Pre-treatment x Treatment
All rats	F(1,37) = 10.47; ##*p* = 0.003	F(1,37) = 10.44; *p* = 0.003	F(1,37) = 1.06; *p* = 0.311	F(1,37) = 0.01; *p* = 0.978
	Two-way ANOVA: F (DFn, DFd), p value	**Pre-treatment (Veh vs. LTZ)**	**Treatment (SHAM vs. ECS)**	**Pre-treatment x Treatment**
	Male rats	F (1,21) = 5.09; $*p* = 0.035	F (1,21) = 0.38; *p* = 0.543	F(1,21) = 0.01; *p* = 0.920
	Female rats	F (1,16) = 26.07; $$$*p* < 0.001	F (1,16) = 3.89; *p* = 0.066	F (1,16) = 0.22; *p* = 0.647

Figure 2. Immobility (s)

Three-way ANOVA: F (DFn, DFd), *p* value	Sex	Pre-treatment (Veh vs. LTZ)	Treatment (SHAM vs. ECS)	Sex x Pre-treatment x Treatment
**A.** PND 52	F(1,52) = 1.75; *p* = 0.192	F(1,52) = 0.55; *p* = 0.463	F(1,52) = 49.47; *p* < 0.001	F(1,52) = 9.81; *p* = 0.003
**B.** PND 54	F(1,51) = 6.00; #*p* = 0.018	F(1,51) = 0.08; *p* = 0.782	F(1,51) = 6.06; *p* = 0.017	F(1,51) = 2.62; *p* = 0.112
**C.** PND 58	F(1,50) = 14.49; ###*p* < 0.001	F(1,50) = 1.11; *p* = 0.297	F(1,50) = 7.73; *p* = 0.008	F(1,50) = 1.19; *p* = 0.280
	Two-way ANOVA: F (DFn, DFd), p value	**Pre-treatment (Veh vs. LTZ)**	**Treatment (SHAM vs. ECS)**	**Pre-treatment x Treatment**
	**B.** Male rats - PND 54	F (1,29) = 2.66; *p* = 0.114	F (1,29) = 5.24; *p* = 0.030	F (1,29) = 2.08; *p* = 0.160
	Female rats - PND 54	F(1,22) = 2.55; *p* = 0.124	F(1,22) = 1.66; *p* = 0.211	F(1,22) = 9.68; *p* = 0.005
	**C.** Male rats - PND 58	F(1,28) = 0.74; *p* = 0.398	F(1,28) = 0.01; *p* = 0.935	F(1,28) = 0.01; *p* = 0.962
	Female rats - PND 58	F(1,22) = 0.42; *p* = 0.525	F(1,22) = 14.49; *p* = 0.001	F(1,22) = 2.02; *p* = 0.169

Figure 2. Climbing (s)

Three-way ANOVA: F (DFn, DFd), *p* value	Sex	Pre-treatment (Veh vs. LTZ)	Treatment (SHAM vs. ECS)	Sex x Pre-treatment x Treatment
**D.** PND 52	F(1,52) = 2.64; *p* = 0.111	F(1,52) = 0.82; *p* = 0.370	F(1,52) = 47.82; *p* < 0.001	F(1,52) = 8.41; *p* = 0.006
**E.** PND 54	F(1,51) = 7.00; #*p* = 0.011	F(1,51) = 0.35; *p* = 0.556	F(1,51) = 6.10; *p* = 0.017	F(1,51) = 2.37; *p* = 0.130
**F.** PND 58	F(1,50) = 9.80; ##*p* = 0.003	F(1,50) = 0.61; *p* = 0.437	F(1,50) = 7.55; *p* = 0.008	F(1,50) = 0.67; *p* = 0.418
	Two-way ANOVA: F (DFn, DFd), p value	**Pre-treatment (Veh vs. LTZ)**	**Treatment (SHAM vs. ECS)**	**Pre-treatment x Treatment**
	**E.** Male rats - PND 54	F (1,29) = 0.77; *p* = 0.386	F (1,29) = 4.73; *p* = 0.038	F (1,29) = 2.47; *p* = 0.127
	Female rats - PND 54	F(1,22) = 2.31; *p* = 0.143	F(1,22) = 1.89; *p* = 0.183	F(1,22) = 11.20; *p* = 0.003
	**F.** Male rats - PND 58	F(1,28) = 0.91; *p* = 0.349	F(1,28) = 0.21; *p* = 0.649	F(1,28) = 0.06; *p* = 0.814
	Female rats - PND 58	F(1,22) = 0.07; *p* = 0.800	F(1,22) = 8.81; *p* = 0.007	F(1,22) = 1.32; *p* = 0.262

Figure 2. Swimming (s)

Three-way ANOVA: F (DFn, DFd), *p* value	Sex	Pre-treatment (Veh vs. LTZ)	Treatment (SHAM vs. ECS)	Sex x Pre-treatment x Treatment
**G.** PND 52	F(1,52) = 0.05; *p* = 0.817	F(1,52) = 0.02; *p* = 0.887	F(1,52) = 12.18; p = 0.001	F(1,52) = 3.53; *p* = 0.066
**H.** PND 54	F(1,51) = 0.51; *p* = 0.477	F(1,51) = 1.69; *p* = 0.199	F(1,51) = 0.06; *p* = 0.812	F(1,51) = 0.08; *p* = 0.779
**I.** PND 58	F(1,50) = 3.75; *p* = 0.059	F(1,50) = 0.06; *p* = 0.809	F(1,50) = 0.07; *p* = 0.788	F(1,50) = 3.40; *p* = 0.071

Supplementary Table S2. Statistical analysis during adulthood. This table represents the statistical analysis performed for each one of the figures shown in the manuscript. Green-shadow boxes represent statistically significant effects.

Adulthood

Figure 3. Barnes maze - Time (s)

Three-way ANOVA: F (DFn, DFd), p value	**Sex**	**Pre-treatment (Veh vs. LTZ)**	**Treatment (SHAM vs. ECS)**	**Sex x Pre-treatment x Treatment**
**A.** PND 89 -T1	F(1,52) = 12.24; ###p = 0.001	F(1,52) = 1.79; p = 0.187	F(1,52) = 4.15; p = 0.047	F(1,52) = 0.05; p = 0.828
**B.** PND 89 - T2	F(1,52) = 12.29; ###p < 0.001	F(1,52) = 0.01; p = 0.860	F(1,52) = 0.33; p = 0.566	F(1,52) = 2.56; p = 0.116
**C.** PND 89 - T3	F(1,52) = 10.41; ##p = 0.002	F(1,52) = 0.31; p = 0.581	F(1,52) = 0.49; p = 0.487	F(1,52) = 0.26; p = 0.615
**D.** PND 89 - test	F(1,52) = 9.49; ##p = 0.003	F(1,52) = 3.72; p = 0.059	F(1,52) = 0.14; p = 0.709	F(1,52) = 0.47; p = 0.495
**E.** PND 90 - re-test	F(1,52) = 6.65; #p = 0.013	F(1,52) = 0.01; p = 0.907	F(1,52) = 4.24; p = 0.045	F(1,52) = 0.95; p = 0.335
**F.** PND 96 - T1	F(1,52) = 5.60; #p = 0.022	F(1,52) = 0.23; p = 0.630	F(1,52) = 4.64; p = 0.036	F(1,52) = 0.75; p = 0.392
**G.** PND 96 - test	F(1,52) = 4.43; #p = 0.040	F(1,52) = 0.18; p = 0.669	F(1,52) = 1.32; p = 0.255	F(1,52) = 0.31; p = 0.581

Two-way ANOVA: F (DFn, DFd), p value	**Pre-treatment (Veh vs. LTZ)**	**Treatment (SHAM vs. ECS)**	**Pre-treatment x Treatment**
**A.** Male rats - PND 89 - T1	F (1,29) = 3.01; p = 0.093	F (1,29) = 0.80; p = 0.379	F (1,29) = 1.11; p = 0.300
Female rats - PND 89 - T1	F (1,23) = 0.01; p = 0.945	F (1,23) = 6.39; p = 0.019	F (1,23) = 4.01; p = 0.057
**B.** Male rats - PND 89 - T2	F (1,29) = 0.61; p = 0.441	F (1,29) = 0.71; p = 0.407	F (1,29) = 1.56; p = 0.222
Female rats - PND 89 - T2	F (1,23) = 4.78; p = 0.039	F (1,23) = 0.28; p = 0.600	F (1,23) = 3.23; p = 0.085
**C.** Male rats - PND 89 - T3	F (1,29) = 1.11; p = 0.302	F (1,29) = 1.48; p = 0.233	F (1,29) = 0.68; p = 0.415
Female rats - PND 89 -T3	F (1,23) = 1.08; p = 0.310	F (1,23) = 1.06; p = 0.314	F (1,23) = 0.37; p = 0.550
**D.** Male rats - PND 89 - test	F (1,29) = 3.13; p = 0.088	F (1,29) = 7.46; p = 0.011	F (1,29) = 0.60; p = 0.444
Female rats - PND 89 - test	F (1,23) = 0.96; p = 0.336	F (1,23) = 6.26; p = 0.020	F (1,23) = 0.03; p = 0.855
**E.** Male rats - PND 90 - re-test	F (1,29) = 0.01; p = 0.927	F (1,29) = 7.17; p = 0.012	F (1,29) = 0.12; p = 0.724
Female rats - PND 90 - re-test	F (1,23) = 0.17; p = 0.688	F (1,23) = 0.06; p = 0.804	F (1,23) = 2.09; p = 0.162
**F.** Male rats - PND 96 - T1	F (1,29) = 0.34; p = 0.536	F (1,29) = 7.74; p = 0.009	F (1,29) = 0.88; p = 0.356
Female rats - PND 96 - T1	F (1,23) = 0.04; p = 0.844	F (1,23) = 0.79; p = 0.382	F (1,23) = 0.03; p = 0.864
**G.** Male rats - PND 96 - test	F (1,29) = 0.18; p = 0.676	F (1,29) = 3.61; p = 0.067	F (1,29) = 0.16; p = 0.696
Female rats - PND 96 - test	F (1,23) = 0.04; p = 0.853	F (1,23) = 0.12; p = 0.733	F (1,23) = 0.17; p = 0.687

Supplementary Figure S1. Barnes maze - Number of errors

Three-way ANOVA: F (DFn, DFd), p value	**Sex**	**Pre-treatment (Veh vs. LTZ)**	**Treatment (SHAM vs. ECS)**	**Sex x Pre-treatment x Treatment**
**A.** PND 89 -T1	F(1,52) = 0.08; p = 0.776	F(1,52) = 0.27; p = 0.606	F(1,52) = 1.64; p = 0.207	F(1,52) = 0.60; p = 0.443
**B.** PND 89 - T2	F(1,52) = 2.13; p = 0.151	F(1,52) = 1.14; p = 0.290	F(1,52) = 0.27; p = 0.603	F(1,52) = 5.79; p = 0.020
**C.** PND 89 - T3	F(1,52) = 1.42; p = 0.239	F(1,52) = 0.42; p = 0.522	F(1,52) = 0.02; p = 0.882	F(1,52) = 0.38; p = 0.541
**D.** PND 89 - test	F(1,51) = 1.72; p = 0.196	F(1,51) = 0.90; p = 0.347	F(1,51) = 0.07; p = 0.786	F(1,51) = 0.52; p = 0.474
**E.** PND 90 - re-test	F(1,52) = 0.60; p = 0.443	F(1,52) = 0.28; p = 0.600	F(1,52) = 4.83; p = 0.033	F(1,52) = 0.73; p = 0.398
**F.** PND 96 - T1	F(1,52) = 0.32; p = 0.576	F(1,52) = 0.65; p = 0.425	F(1,52) = 0.01; p = 0.988	F(1,52) = 0.65; p = 0.423
**G.** PND 96 - test	F(1,52) = 0.21; p = 0.650	F(1,52) = 0.14; p = 0.705	F(1,52) = 0.28; p = 0.598	F(1,52) = 1.87; p = 0.178

Figure 4. FST - PND 101

Three-way ANOVA: F (DFn, DFd), p value	**Sex**	**Pre-treatment (Veh vs. LTZ)**	**Treatment (SHAM vs. ECS)**	**Sex x Pre-treatment x Treatment**
**A.** Immobility (s)	F(1,50) = 0.18; p = 0.671	F(1,50) = 1.38; p = 0.247	F(1,50) = 7.31; p = 0.009	F(1,50) = 0.43; p = 0.513
**B.** Climbing (s)	F(1,50) = 1.17; p = 0.285	F(1,50) = 0.51; p = 0.478	F(1,50) = 9.65; p = 0.003	F(1,50) = 0.45; p = 0.504
**C.** Swimming (s)	F(1,50) = 2.02; p = 0.161	F(1,50) = 2.11; p = 0.153	F(1,50) = 0.05; p = 0.817	F(1,50) = 0.01; p = 0.908

Supplementary Figure S1. Evaluating the long-term effects on cognitive performance in adulthood following adolescent ECS and/or letrozole treatments in male and female rats. Number of errors performed in **(A-C)** 3 consecutive trials (T1, T2, T3) and **(D-E)** 2 test sessions (spaced 24 h, test on PND 89 and re-test on PND 90) to complete the Barnes maze. Number of errors performed in **(F)** a new trial (changing the location of the target box on PND 96-T1) and **(G)** test session (PND 96) to complete the Barnes maze a week after. Data represents mean ± SEM of the number of errors performed while completing the Barnes maze. Individual values are shown for each rat (symbols). Three-way ANOVAs (independent variables: Sex, Pre-Treatment and Treatment) or two-way ANOVAs (independent variables: Pre-Treatment and Treatment) were performed and results are shown in Supplementary Table S2. Veh: vehicle; LTZ: letrozole; ECS: electroconvulsive seizures; S: SHAM; PND: post-natal day.

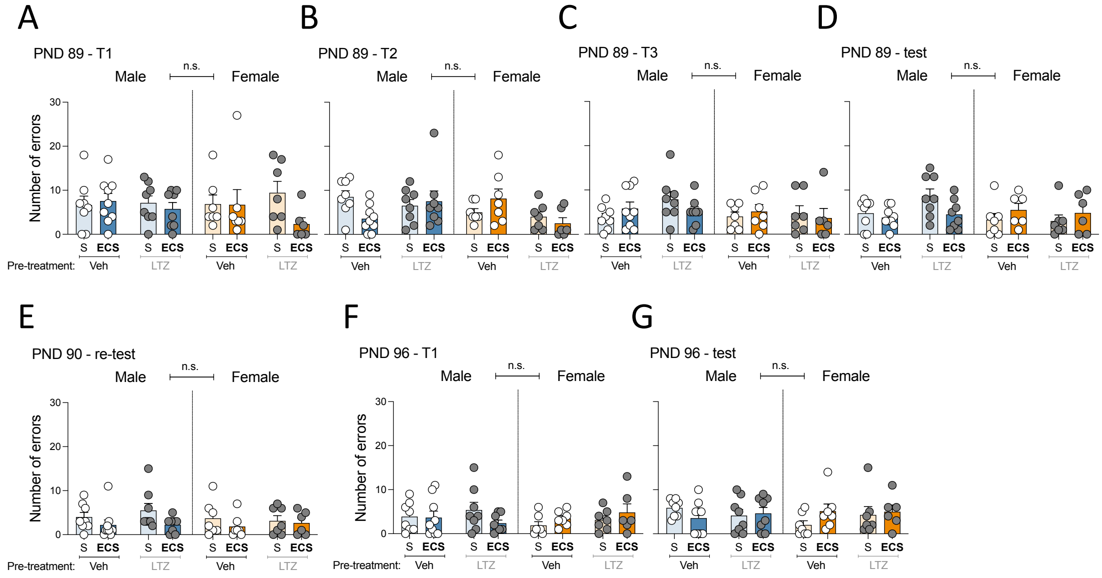

Supplementary Figure S2. Monitoring the different strategies used to resolve the Barnes maze test. Type of strategy used to resolve the Barnes maze in adulthood following adolescent treatment. Data represents the % strategy used to resolve the maze (direct, serial, mixed or not completed) for each treatment group. Veh: vehicle; LTZ: letrozole; ECS: electroconvulsive seizures; S: SHAM.

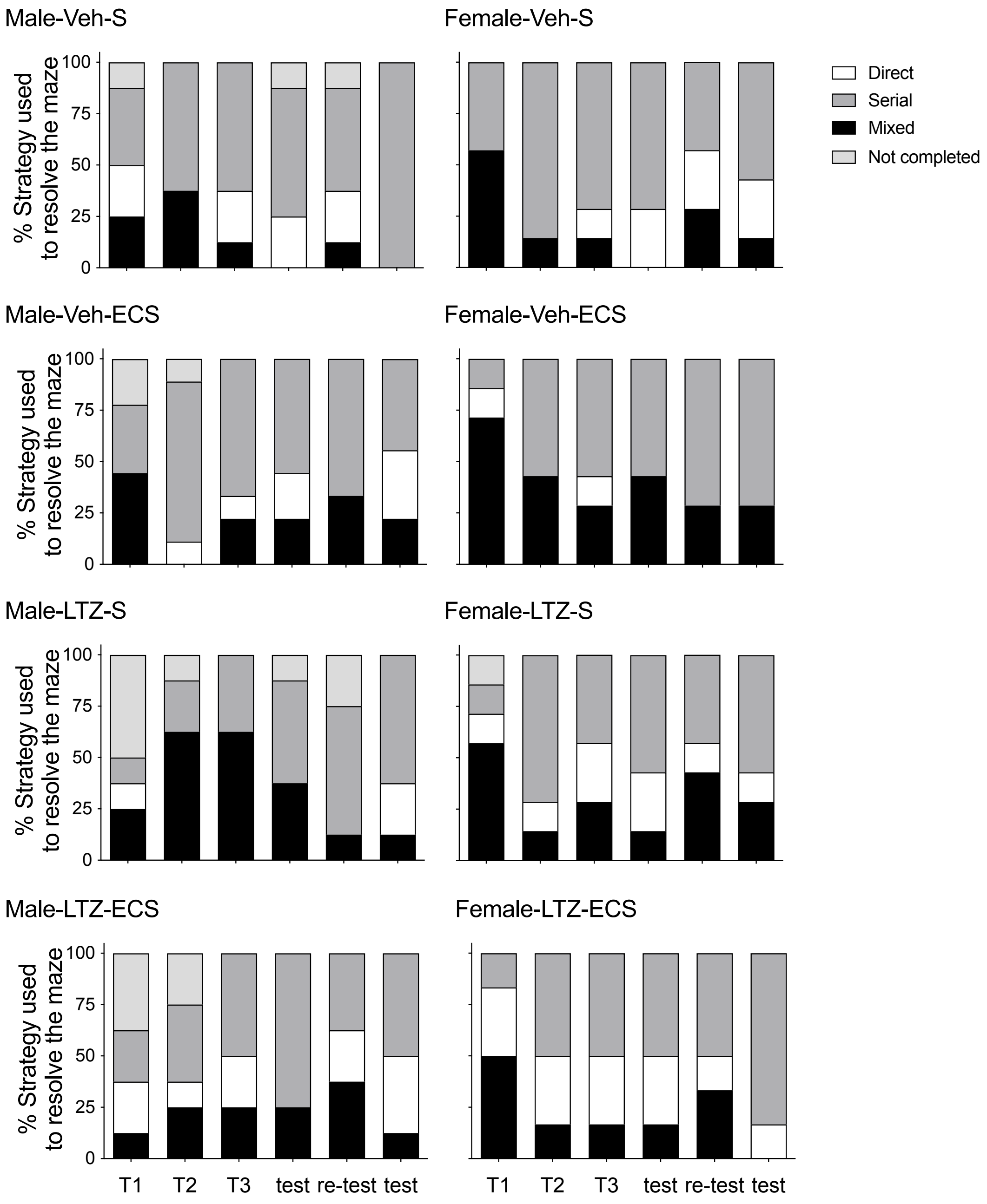

Supplementary Figure S3. Monitoring the impact of the adolescent treatment on affective-like behavior across time in male and female rats. Time (s) spent immobile across time after ECS and/or letrozole exposure in male and female rats. Data represents mean ± SEM of the time (s) spent in immobile. Veh: vehicle; LTZ: letrozole; ECS: electroconvulsive seizures; PND; post-natal day.

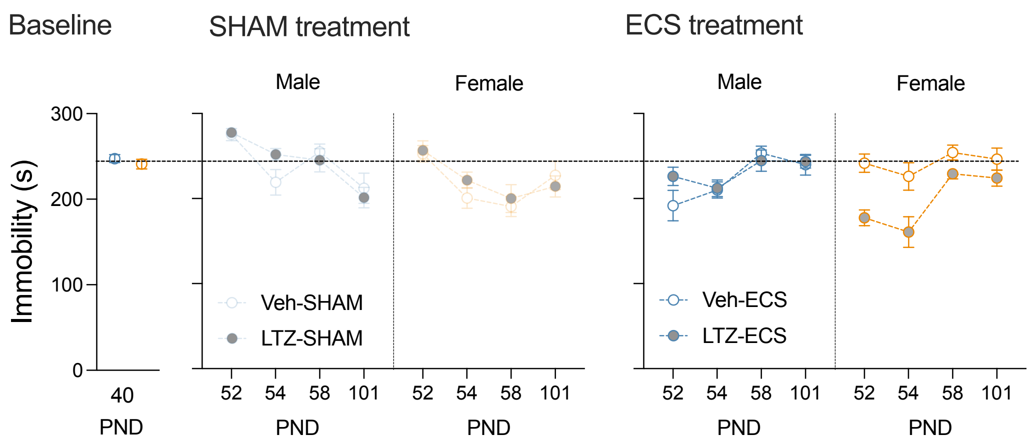

Supplement: pyad047_suppl_Supplementary_Materials [file pyad047_suppl_supplementary_materials.pdf]
